# Subcutaneous interleukin-2, interferon alpha-2b and 5-fluorouracil in metastatic renal cell carcinoma as second-line treatment after failure of previous immunotherapy: a phase II trial

**DOI:** 10.1038/sj.bjc.6601419

**Published:** 2003-12-09

**Authors:** A Ravaud, N Trufflandier, J M Ferrière, M Debled, J Palussière, L Cany, R Gaston, S Mathoulin-Pélissier, B N Bui

**Affiliations:** 1 Department of Medicine, Institut Bergonié, Bordeaux, France; 2 Department of Urology, University Hospital, Bordeaux, France; 3 Department of Radiology, Institut Bergonié, Bordeaux, France; 4 Department of Surgery, Institut Bergonié, Bordeaux, France; 5 Department of Urology, Clinique Saint Augustin, Bordeaux, France; 6 Department of Biostatistics, Institut Bergonié, Bordeaux, France

**Keywords:** immunotherapy, interleukin-2, interferon alpha, 5-fluorouracil, renal cell carcinoma, second-line treatment

## Abstract

The association of interleukin-2 (IL-2), interferon alpha-2a (IFN*α*), 5-fluorouracil (5-FU) has been reported to induce response in metastatic renal cell carcinoma (MRCC). This study evaluated IL-2, IFN*α* and 5FU as second-line treatment after failure under immunotherapy. A total of 35 patients received IL-2, at 9 × 10^6^ IU m^−2^, once or t.i.d, 5 days a week, every other week. Interferon alpha was administered at 6 MUI, TIW along with IL-2 every week. 5-Fluorouracil was given at 750 mg m^−2^ day^−1^ on days 1–5 every 4 weeks. One cycle lasted 8 weeks. All patients were evaluable for response and toxicity. There were two objective responses (5.7%) and 14 stable diseases (40%). Survival was 14 months. In all, 17 patients experienced grade 3 toxicity. The predictive factor for progression to second-line immunotherapy was the results of first-line immunotherapy, and performance status, delay from primary tumour to metastases and response or stabilisation to chemo-immunotherapy for survival. IL-2, IFN*α* and 5-FU induce low objective response but stabilisation in patients with MRCC having failed with immunotherapy, and may be considered only in selected patients on performance status, stabilisation or response after first-line immunotherapy and interval from their primary tumour to metastases.

The prognosis of patients with metastatic renal cell carcinoma (MRCC) remains poor with an estimated 5-year survival of 0–20% (Linehan *et al*, 2001). Interferon alpha (IFN*α*) and interleukin-2 (IL-2) have shown objective responses in 10–25% of patients (Rosenberg *et al*, 1989; Atzpodien *et al*, 1993; Buter *et al*, 1993; Fyfe *et al*, 1995; Savage and Muss, 1995; Yang and Rosenberg, 1997; Négrier *et al*, 1998) and long-lasting responders (Fyfe *et al*, 1996). More recently, INF*α* has been shown to prolong survival compared to hormonotherapy (Medical Research Council Renal Cancer Collaborators, 1999), although progression occurred in most patients.

When this study was designed, no effective second-line treatment arrising from chemotherapy (Yagoda *et al*, 1995) or cellular therapy (Figlin *et al*, 1998) was available. Only one study in 13 patients with MRCC treated with s.c. IL-2 after failure under IFN*α* reported four partial responses (Lissoni *et al*, 1992).

Based on preclinical data suggesting a synergism between IL-2 and 5-fluorouracil (5-FU) on one hand and IFN*α* and 5-FU on the other, added to the well-known synergism of the association of IL-2 and IFN*α* (Cameron *et al*, 1988), the combination of IL-2, IFN*α* and 5-FU has been investigated in renal cell carcinoma. In animal experiments, IL-2 potentiates the antitumoral activity of various cytotoxic drugs including 5-FU (Gauny *et al*, 1989; Kawano *et al*, 1994; Lee *et al*, 1994). Modulation of 5-FU with IFN*α* has been more extensively studied. IFN*α* induces thymidine phosphorylase, enhancing the conversion of 5-FU to the active 5-fluorodeoxyuridine monophosphate (FdUMP) inducing the depletion of thymidine triphosphate pools and DNA breakpoint, leading so far to an increase of the cytoxicity of 5-FU (Wadler *et al*, 1990; Morita and Tokue, 1999). Moreover, IFN*α* inhibits the intracellular uptake of thymidine (Pfeffer and Tamm, 1984), and thymidilate synthase (Elias and Sandoval, 1989). At the beginning of the 1990s, clinical trials reported an increased response rate when 5-FU was added to IL-2 and IFN*α* (Atzpodien *et al*, 1993; Hofmockel *et al*, 1996; Joffe *et al*, 1996; Ellerhorst *et al*, 1997). Groups using an identical schedule as Atzpodien *et al* showed the response rate as ranging from 16 to 48% (Atzpodien *et al*, 1993; Hofmockel *et al*, 1996; Joffe *et al*, 1996; Ellerhorst *et al*, 1997).

Owing to the lack of a validated second-line treatment after immunotherapy in MRCC, the possibility that a second-line chemo-immunotherapy might prevent progression under previous immunotherapy was tested. Based on the reported higher response rate when 5-FU was added to IL-2 and IFN*α*, this study was designed to test the ability of the association of IL-2, IFN*α* and 5-FU to induce an objective response or at least stabilization while patients had progressed under IFN*α* and/or IL-2.

## PATIENTS AND METHODS

### Patients

Eligible patients had histologically proven renal cell carcinoma with progressive metastatic disease after a previous immunotherapy with IFN*α* and/or IL-2. Patients were adults less than 75 years of age, who had a Karnofsky performance status >70%. Patients were required to have measurable metastatic disease. They were not to have received either immunotherapy or radiotherapy in the previous 4 weeks. Adequate organ functions were required without cardiac, respiratory, hepatic, renal, neurologic or psychiatric disorders. They had normal blood cell counts, normal bilirubin level, creatinine concentrations less than 180 *μ*mol/l^−1^, normal cardiac function and a life expectancy of at least 3 months. Patients with severe infection, known positivity of human immunodeficiency virus test or chronic hepatitis were excluded, as were patients on corticosteroids. Patients did not have history of an organ allograft or other malignancies. Pregnant or lactating women were also excluded.

The trial was approved by the CCPPRB in Bordeaux according to the French law. The study was conducted according to the principles of Good Clinical Practice.

### Pretreatment evaluation

In addition, clinical history and physical examination were recorded for all patients. Preinclusion staging included cerebral, thoracic, abdominal CT scans and a bone scan. Written informed consent was obtained before inclusion in the trial.

### Treatment plan (Table 1)

Interleukin-2 (Proleukin; Chiron Therapeutics, Suresnes, France) was given subcutaneously at a dose of 9 × 10^6^ IU m^−2^, twice on days 1 and 2, once a day on days 3–5 and every other week for 8 weeks. Interferon alpha (Introna; Schering Plough, Levallois-Perret, France) was administered at a dose of 6 × 10^6^ IU, three times a week, during weeks with IL-2. 5-Fluorouracil was delivered by a continuous infusion at 750 mg m^−2^ day^−1^ for five consecutive days every 4 weeks, starting with IL-2 and IFN*α* in the first week. Each time an objective response or a stable disease occurred, an additional identical course of treatment was given after 1 week's rest. Table 1

**Table 1:** Schedule and dose of treatment

IL-2	*	*	⋄	⋄	⋄	*	*	⋄	⋄	⋄	
IFN*α*	#		#		#	#		#		#	
5-FU	φ	φ	φ	φ	φ						
day	1	2	3	4	5	15		17		19	29
											Second cycle

* =IL-2: 9 × 10^6^ IU m^−2^ × 2, ⋄=IL-2: 9 × 10^6^ IU m^−2^, #=IFN*α*: 6 × 10^6^ IU, φ=5-FU 750 mg m^−2^.

### Evaluation of treatment

Evaluation of tumour response, including thoracic and abdominal CT-scan and a bone scan, was performed every 8 weeks of treatment. The World Health Organization (WHO) criteria were used to determine tumour response (Miller *et al*, 1981). Complete response (CR) was defined as the complete disappearance of all measurable and evaluable tumour sites for at least 4 weeks. The duration of CR was calculated from the first date of documentation of CR to the date of the first evaluation of disease progression. Partial response (PR) was considered to be a ⩾50% decrease in the sum of products of the greatest perpendicular diameters lasting for at least 4 weeks, with no increase in known lesions and without appearance of any new lesions. When the evaluation showed a <50% decrease in lesions or a <25% increase, patients were considered to have a stable disease (SD). The duration of PR and SD was calculated from the first day of treatment. Progressive disease (PD) was considered to be when any lesion increased by ⩾25% or when a new lesion appeared. The results of the successive bone scans were considered as PD in the case of appearance of new spots, stable if not, and complete regression only if all spots disappeared. Patients who presented with a CR, PR or SD were evaluated every 2–3 months during the first year and then every 4–6 months.

Survival duration was evaluated from the start of treatment to the date of the last contact or the date of death. Progression-free survival was calculated from the start of treatment to the date of last follow-up or the date of progression.

Toxicities encountered were classified according to the WHO grading system.

### Statistical analysis

The primary end point was the response rate. The secondary end points were stabilisation rate, prolonged stabilisation rate (at least a period following chemo-immunotherapy as long as two cycles of treatment: 2 × [2 × 8 weeks]=32 weeks or 8 months), overall survival, toxicity and prognostic factors for progression under second-line chemo-immunotherapy and overall survival.

The trial was conducted according to the two-stage Gehan design (Gehan, 1961). As first-line treatment, IFN*α* and IL-2 have shown objective responses in 18% of patients included into trials that we conducted (Négrier *et al*, 1998; Ravaud *et al*, 1994); we planned to detect a response rate ⩾10%. We assessed the response rate after 29 patients had been recruited to have a 95% chance of detecting at least one response when the actual response rate was ⩾10%. If at least one response occurred in the first 29 patients, we planned to increase the number of patients to assess the response rate with 5% precision (i.e., one response justified the inclusion of four more patients: at least 33 patients for the study).

To study prognostic factors, patients presenting response and stabilisation were pooled. Progressive disease was considered in the case of progression of the disease at tumour evaluation performed at 8 weeks. The following potential clinical prognostic parameters were analysed: gender, time from primary tumor to occurrence of metastases (<12 months *vs* ⩾12 months), type of first-line immunotherapy (IL-2 *vs* IFN*α* alone), response to first-line immunotherapy (PD *vs* objective response or stabilisation), number of sites before second-line treatment (1 *vs* >1) and general status (Karnofsky ⩾90% *vs* <90%). The association between response and stabilisation after second-line immunotherapy was assessed using the *χ*^2^ test and survival distribution was estimated using the Kaplan–Meier method (Kaplan and Meier, 1958). The relationship between survival and parameters was analysed with the log-rank test (Mantel, 1966). Parameters that were significantly associated with survival at a *P*-value of <0.10 were included in a forward stepwise Cox model (Mantel, 1966).

## RESULTS

### Patient characteristics

From September 1994 to April 2000, 35 patients with MRCC were entered into the trial. All patients were evaluable for response and for toxicity and their main characteristics are outlined in Table 2

**Table 2:** Characteristics of patients

**Characteristics**	**No.**	**%**
Eligible patients	35	
*Patients assessable*		
for toxicity	35	100
for response	35	100
Men/women	27/8	77/23
Age (years) median (range)	62 (25–75)	
		
*Performance status (Karnofsky)*		
100%	7	20
90%	14	40
80%	12	34.3
70%	2	5.7
		
*Time from diagnosis to first metastases*		
<12 months	25	71.4
⩾12 months	10	28.6
Prior nephrectomy	35	100
		
*Prior immunotherapy*		
IFN	21	60
IL-2	9	25.7
IL-2+IFN	5	14.3
		
*Response to first-line immunotherapy*		
IFN (CR, PR, SD, PD)	1, 2, 12, 6	
IL-2 (CR, PR, SD, PD)	0, 1, 4, 4	
IL-2+IFN (CR, PR, SD, PD)	0, 2, 2, 1	
		
*Site of metastatic disease*		
Lung	27	77.1
Mediastinal lymph nodes	14	40
Bone	6	17
Abdominal lymph nodes	5	14
Liver	4	11
Recurrence at nephrectomy site	3	9
Others	8	23
		
*Number of sites with metastases*		
1	9	25.7
>1	26	74.3

IFN=interferon, IL-2=interleukin-2, CR=complete response, PR=partial response, SD=stable disease, PD=progressive disease.

. Most patients had an ambulatory performance status: 21 patients (60%) had a Karnofsky performance status ⩾90%, while 14 (40%) patients had a Karnofsky performance status <90%. The time from diagnosis of renal cell carcinoma to the occurrence of metastases was less than 12 months in 25 patients (71.4%). All patients had a prior nephrectomy. The median delay from diagnosis of metastatic disease to first immunotherapy was 3.4 months (range: 0–29 months). For the first-line immunotherapy, 21 patients were treated with IFN*α*, nine with IL-2 and five with the association of IFN*α* and IL-2. Six patients (17.2%) showed an objective response (1 CR and 5 PR), while 18 (51.4%) had a SD. In a median time of 9 months (range: 2–77 months), patients were included in this study. At the time of second-line treatment, the metastatic disease was localised in the lung (27 patients), mediastinal or abdominal lymph nodes (17 patients), bone (six patients), liver (four patients) and at the nephrectomy site (three patients). In all, 26 patients (74%) had at least two tumour sites at the time of second-line treatment initiation.

### Administration of treatment and toxicity

During cycles, the median dose given to patients was 100% for each drug. The main toxicities (Table 3

**Table 3:** Toxicity

	**Number of patients (%) with toxicities by WHO grades I, II, III and IV**			
	**I (%)**	**II (%)**	**III %)**	**IV (%)**
Decrease in performance status	8 (23)	17 (49)	3 (9)	0
Fever	1 (3)	17 (49)	6 (17)	0
Diarrhoea	2 (6)	8 (23)	2 (6)	0
Nausea/vomiting	5 (14)	8 (23)	4 (11)	0
Local skin pain	0	2 (6)	0	0
General skin disorders	5 (14)	5 (14)	1 (3)	0
Hypotension	1 (3)	4 (11)	3 (9)	0
Mucositis	3 (9)	0	2 (6)	0
Cardiotoxicity	0	0	2 (6)	0
Neurological	0	1 (3)	0	0
Psychiatric	0	3 (9)	0	0
Infection	0	2 (6)	0	0
Weight gain	1 (3)	0	0	0
Haematological	5 (14)	4 (11)	2 (6)	0
Increase in transaminases	2 (6)	0	0	0
Hypercreatininaemia	2 (6)	0	0	0
Others	0	4 (11)	1 (3)	0

) were decrease in performance status (28 patients, 80%), fever (24 patients, 69%), nausea/vomiting (17 patients, 49%), diarrhoea (12 patients, 34%), cutaneous erythema (11 patients, 31%), hypotension (eight patients, 23%) and haematological disturbances (11 patients, 31%). In all, 17 patients (49%) had treatment-related grade 3 toxicity; 27 grade 3 events were reported: fever (six patients, 17%), nausea/vomiting (four patients, 11%), decrease in performance status, hypotension (three patients, 9%), diarrhoea, cardiotoxicity, mucositis (two patients) and skin erythema, anaemia, neutropenia and hypothyroidism (one patient). Nevertheless, none had grade 4 toxicity or died within the treatment course.

### Response to treatment and survival

**Figure 1:**
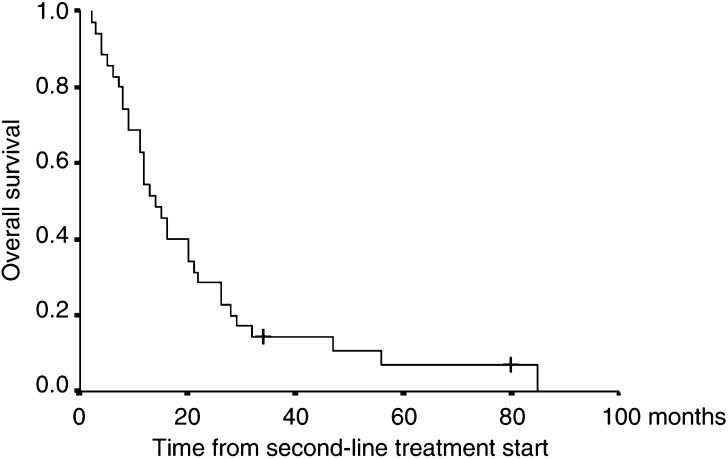
Overall survival.

The median follow-up was 14 months. At the evaluation performed after 8 weeks of treatment, two patients (5.7%; 95% CI: 0.07–19.15%) had achieved an objective response, with one CR obtained after an immediate subsequent mediastinal radiotherapy. The duration of response was 6 and 56+ months. In all, 14 patients (40%; 95% CI: 23.8–57.9%) had SD for a median time of 4 months (range: 2–16 months), including four patients (11.4%) with SD >8 months, while 19 showed disease progression. The sites of response were lung and lymph nodes for both patients. The median survival of all patients was 14 months (95% CI: 10.4–17.7 months) (Figure 1

). Patients who showed stabilisation or an objective response had a median survival of 22 months (95% CI: 10.2–33.8 months), while those with a PD had a median survival of 9 months (95% CI: 4.7–13.3 months).

### Predictive factors for progression to second-line immunotherapy

In univariate analysis, only the results of primary immunotherapy: stabilisation or objective response *vs* progression were significantly predictive for PD under second-line immunotherapy treatment (*χ*^2^, *P*: 0.026). Neither gender, time from primary tumour to occurrence of metastases (<12 months *vs* ⩾12 months), type of first-line immunotherapy (IL-2-based treatment *vs* IFN*α* alone), number of sites before second-line treatment (1 *vs* >1) nor general status (Karnofsky ⩾90 *vs* <90%) reached statistical significance.

### Predictive factors for survival following second-line immunotherapy

In univariate analysis, factors significantly (*P*<0.05) associated with better survival were objective response or stabilisation after second-line treatment (*P*<0.001) and general status at the time of second-line treatment (*P*<0.01). Neither the type of first-line immunotherapy nor the number of sites at second-line treatment was predictive of outcome.

Parameters showing an association with survival in univariate analysis with a degree of significance <0.10 were included in a forward stepwise Cox mutivariate analysis. General performance status (Karnofsky ⩾90% *vs* <90%) (*P*: 0.003), time from primary tumour to metastases (*P*: 0.033) and response or stabilisation to second-line treatment (*P*: 0.038) were considered as independent factors predictive of survival (Table 4

**Table 4:** Univariate and multivariate stepwise Cox model analysis of survival for all patients

	**Univariate**	**Multivariate**		
**Prognostic factor**	***P***	**HR**	**95% CI**	***P***
Objective response and stabilisation to second–line immunotherapy with IL-2, IFN*α* and 5-FU	0.006	2.43	1.05–5.6	0.038
General performance status (Karnofsky ⩾90 *vs* <90%)	0.007	3.72	1.55–8.91	0.003
Time interval from primary tumour to occurrence of metastases (⩽12 *vs* >12 months)	0.07	2.71	1.08–6.78	0.033

).

## DISCUSSION

The RR obtained in this study after a second-line immunotherapy-based treatment remains low (two out of 35 patients) despite one prolonged complete remission (>56 months). Since the start of the study, only two other studies have examined this theme (Paolorossi *et al*, 1995; Escudier *et al*, 1999). A large study in 113 patients confirmed the low response (four patients, 3.5%) and stabilisation (13 patients, 11.5%) rates, following a switch from IFN*α* to IL-2 and from IL-2 to IFN*α* after failure of the first-line cytokine therapy (Escudier *et al*, 1999). In the former study (Escudier *et al*, 1999), none of the patients received the association of both cytokines after one had already failed. In another study (Paolorossi *et al*, 1995) following the initial work of Lissoni *et al* (1992), 15 patients received IFN*α* and vinblastine after failure under IL-2. Two patients showed an objective response and five had stabilisation (Paolorossi *et al*, 1995).

Nevertheless, the occurrence of stabilisation in these circumstances, second-line treatment in patients, especially while they are still in a good general performance status (Karnofsky ⩾90%) and when no alternative second-line treatment for renal cell carcinoma is available other than inclusion in clinical trials, could be considered to be of clinical interest. Furthermore, in this study, those patients with stabilisation or an objective response had a prolonged median survival of 22 months even after a second-line treatment. Nevertheless, spontaneous slow progression and/or long stabilisation of renal cell carcinoma without any antitumoral treatment may affect interpretation of survival outside compared phase III clinical trials. For this reason, it was considered useful when making decisions to point out predictive factors for outcome following a second-line immunotherapy-based treatment. This study shows that the most clinical significant predictive factor for no progressive disease at 8 weeks under second-line chemo-immunotherapy is the efficacy of first-line immunotherapy, which has not been assessed until now. Only one study reported that only patients with SD or transient responders with first-line cytokine treatment were responders (three out of four responders among 113 treated patients) (Escudier *et al*, 1999). In our study, parameters favourably affecting survival were a good general performance status at initiation of second-line treatment (Karnofsky ⩾90%), the delay from primary tumour to metastases (>12 months) and the response to second-line treatment. Previous studies on prognostic factors for survival in MRCC, especially those carried out in patients under immunotherapy, showed both general performance status and delay from primary tumour to metastases to affect survival significantly (Palmer *et al*, 1992; Négrier *et al*, 2002). As the efficacy of second-line immunotherapy-based treatment was significantly correlated to the efficacy of the first-line treatment and as the second-line treatment had a significant impact on survival, it would have been helpful to study the correlation of survival to the first-line treatment, but the study was not designed for this purpose.

While a second-line immunotherapy-based treatment may be considered in selected patients, this study does not provide sufficient evidence that the association of IL-2, IFN*α* and 5-FU and the schedule used are a standard. This protocol is closely related to the schedule designed for other clinical research trials with IL-2, IFN*α* and ±5-FU within the framework of the French Immunotherapy Group (Ravaud *et al*, 1998; Négrier *et al*, 2000). These large studies showed an unexpectedly low response rate (Ravaud *et al*, 1998; Négrier *et al*, 2000) like the present study, compared to more promising results obtained by others (Atzpodien *et al*, 1993; Hofmockel *et al*, 1996; Joffe *et al*, 1996; Ellerhorst *et al*, 1997; Tourani *et al*, 1998; Allen *et al*, 2000; Dutcher *et al*, 2000; Elias *et al*, 2000; van Herpen *et al*, 2000).

Toxicities encountered by patients during this study were moderate as expected with no grade 4 WHO toxicity and 49% of grade 3. Although the study was performed with second-line treatment, the toxicity profile did not show any differences compared to trials performed with first-line treatment (Ravaud *et al*, 1998; Négrier *et al*, 2000).

In conclusion, we achieved 5.7% of objective response and 40% of stabilisation including 11.4% prolonged stabilisation >8 months with IL-2, IFN*α*, 5-FU in patients with renal cell carcinoma in whom previous first-line immunotherapy failed. Therefore, the clinical benefit has to be considered as limited. Nevertheless, second-line immunotherapy may be considered only for selected patients who show either stabilisation or an objective response at evaluation of first-line immunotherapy, who have a good general status and a delay from the primary tumour to metastasis longer than 12 months. The recommended protocol therefore requires further evaluation.
